# Development of a Home-Based Stress Management Toolkit for Dementia Caring Dyads: Protocol for a Pilot Intervention Development and Feasibility Study

**DOI:** 10.2196/43098

**Published:** 2022-12-14

**Authors:** Melissa Harris, Courtney Van Houtven, Susan Hastings

**Affiliations:** 1 Center of Innovation to Accelerate Discovery and Practice Transformation (ADAPT) Durham VA Health Care System Durham, NC United States; 2 School of Nursing Duke University Durham, NC United States; 3 School of Medicine Duke University Durham, NC United States

**Keywords:** dementia, stress, caregiver, dyad, intervention, nonpharmacologic

## Abstract

**Background:**

People living with dementia (PLWD) and their care partners (dementia caring dyads) are at a heightened risk of experiencing stress-related symptoms and conditions. Yet, many dyadic stress management interventions have had limited uptake by health care systems and in the community. An intervention that combines simple, safe, easy-to-use, nonpharmacologic tools (eg, animatronic social pets, weighted blankets and garments, aromatherapy and bright light therapy devices, acupressure, and massage tools) that can be used in the home may be a promising approach to promote stress management among dementia caring dyads.

**Objective:**

The proposed study aims to develop and user test a dyadic toolkit intervention composed of simple, tangible stress management tools for community-dwelling PLWD and their care partners. This study will also explore the feasibility of collecting several stress-related outcome measures to inform measurement selection for future studies.

**Methods:**

A human-centered design (HCD) approach will be used to increase the likelihood of developing an intervention that will be translatable to real-world settings. This study consists of 2 phases. The first phase will address the discover, define, and design stages of HCD using qualitative focus groups with dementia caring dyads (N=12-16 dyads). Dyadic focus groups (3-4 groups anticipated) will be convened to understand participants’ stress experiences and to co-design a stress management toolkit prototype. Rapid qualitative analysis will be used to analyze focus group data. In phase 2, the toolkit prototype will be user tested for 2 weeks in a new sample to address the validation step of HCD. A within-subjects (n=10 dyads), pre-post design will be used with measures of usability (frequency of toolkit use), feasibility (enrollment and withdrawal rates, adverse events/injuries), and acceptability (satisfaction, benefit) collected via questionnaires (at the end of weeks 1 and 2 of user testing) and focus groups (n=3-4 dyads/group at the end of week 2). The feasibility of collecting participant-reported, stress-related outcomes (neuropsychiatric symptoms of dementia, caregiver stress, dyadic relationship strain) and salivary cortisol as a physiologic measure of stress will be assessed at baseline and after user testing.

**Results:**

This study will yield a working prototype of a stress management toolkit for dementia caring dyads, as well as preliminary data to support the feasibility and acceptability of the intervention. User testing will elucidate areas to refine the prototype and provide data to inform preliminary testing of the intervention. As of September 2022, this study has received institutional ethics board approval with phase 1 recruitment anticipated to begin January 2023.

**Conclusions:**

Few interventions have focused on combining simple, safe, low burden tools to promote stress management among community-dwelling dementia caring dyads. By involving families and exploring feasibility and acceptability at the onset of development, this intervention will have greater potential to be implemented and sustained in the future.

**Trial Registration:**

ClinicalTrials.gov NCT05465551; https://clinicaltrials.gov/ct2/show/NCT05465551

**International Registered Report Identifier (IRRID):**

PRR1-10.2196/43098

## Introduction

### Background

Nearly 1 in 9 older Americans are living with dementia, a chronic, progressive disease that affects every aspect of a person’s health and well-being [1]. People living with dementia (PLWD) often live in a heightened state of stress due to changes in how they perceive and respond to the world around them. When their stress threshold is exceeded, distressing behavioral and psychological symptoms (eg, agitation, anxiety, hallucinations, delusions, sleep disturbances) occur [2,3]. The stress process of PLWD is interdependently related to that of their caregivers and care partners [4]. Thus, changes in how PLWD experience and respond to stress can impact the mental and physical well-being of individuals diagnosed *and* their care partners [4-6]. Dyadic interventions focused on improving stress among PLWD and their care partners are paramount to promoting health and well-being among families living with dementia [4,6,7].

Dyadic stress management interventions for PLWD and their care partners often consist of several components and frequent interactions with health care professionals (eg, case management, advanced medical management, psychoeducation, cognitive behavioral training) [8,9]. While such interventions enable a variety of outcomes to be targeted, their complexity can increase burden on care partners who bear primary responsibility for engaging with the intervention. High degrees of burden and cost, exacerbated by the intensive nature of multilevel interventions, have led to limited real-world uptake by health care systems [10,11]. Simple, home-based, dyadic interventions that place minimal burden on care partners are thus needed to reduce stress-experienced families living with dementia.

Stress management tools such as dementia-friendly music devices, social robot pets and dolls, and acupressure and massage tools have been shown to significantly improve stress-related outcomes for PLWD and their care partners [12-15]. Although their effects on stress have not yet been determined, other tools have demonstrated high degrees of safety, feasibility, and acceptability in this population including weighted blankets and garments, prompted journals, aromatherapy, and bright light therapy devices [16-18]. These tools are designed to be used regularly to help the user remain in a healthy, low-stressed state. This is especially important for PLWD who are known to experience a heightened perception of stress and a decreased tolerance of stressful stimuli, which increases their potential for being in an unhealthy state of distress [2]. These tools are hypothesized to prevent distress by increasing social engagement, providing comfort and relaxation, engaging the senses, and connecting users to in-the-moment feelings and surroundings [19-21]. Importantly, all these tools are applicable to everyday situations and are relatively passive in nature, requiring limited supervision and minimal effort to use. By mitigating caregiver burden, such tool-based interventions are expected to have greater acceptability, uptake, and adherence in dyadic contexts compared with more complex interventions. Despite ongoing research on stress management tools, there is a significant knowledge gap in the delivery of *dyadic* stress management interventions to community-dwelling dementia caring dyads. Few studies have combined such tools to best meet the needs of PLWD and their care partners and few tool-based interventions have been designed with ongoing, iterative feedback from this population which will be essential for optimizing delivery and facilitating broad uptake [21,22].

Prior studies have primarily relied on participant-reported outcomes to measure outcomes of stress, and most have focused solely on care partner–specific outcomes. Participant-reported outcomes are useful indicators of psychosocial components of stress, but they can be difficult to collect among PLWD and are subject to inherent risk of bias when completed by proxy report [23,24]. Including biomarkers of stress as outcomes can mitigate limitations of self-report as physiologic stress reactivity is associated with self-reported stress levels among older adults [25]. Physiologic measures of stress may provide a nuanced understanding of intervention efficacy when supplemented with more traditional participant-reported stress outcomes [7]. A multipronged approach to measuring individual and dyadic stress that captures the biopsychosocial nature of the stress process is critical to examining the impact of interventions on dementia caring dyads [7]; however, the feasibility of collecting physiologic measures of stress in this population needs to be determined prior to use in efficacy trials.

The overarching goal of this study is to design a prototype of a dyadic, tangible stress management toolkit with *and* for PLWD and their families [19]. Using a human-centered design (HCD) approach that prioritizes stakeholder engagement at the outset, PLWD and care partners will collaborate with the research team to create the toolkit using 4 key design steps (discover, define, design, and validate) to optimize the intervention for future use (Table 1) [20,26,27].

The *specific aims* of this study are to:

- Aim 1: Develop a prototype of a dyadic stress management toolkit with and for PLWD and their care partners.
- Aim 2: User test the dyadic stress management toolkit intervention with 10 PLWD and their care partners.
- Aim 3: Explore the feasibility of collecting stress-related outcome measures in dyads participating in user testing, including participant-reported outcomes (ie, neuropsychiatric symptoms of dementia, caregiver stress, dyadic relationship strain) and salivary cortisol biospecimens as a physiologic measure of stress.

**Table 1:** Human-centered design steps addressed through study phases 1 and 2.

Phase and aim	Step and definition
Phase 1 (aim 1)	Discover: gather data to understand perceptions, opinions, motivations, experiences, and insights of participants Define: use insights and knowledge discovered to define problem Design: brainstorm, identify, and co-develop potential solutions and protypes with stakeholders
Phase 2 (aims 2 and 3)	Validate: user test developed solutions and prototypes on a small scale with end users to identify refinements and modifications needed to improve prototype

### Preliminary Studies

The first author conducted a within-subjects, pre-post design study (n=21 dyads) to examine the feasibility and acceptability of a virtually delivered weighted blanket intervention for community-dwelling PLWD [28]. Findings showed high degrees of feasibility (enrollment rate=64%, 21 dyads recruited in <4 months, blankets used for the recommended duration 23.8/30 days, SD 6.4, withdrawal rate=5%, no injuries/adverse events) and acceptability (ie, tolerability, satisfaction, benefit) as reported by PLWD and their care partners. This study demonstrated the feasibility and acceptability of 1 potential tool for the toolkit prototype. The first author also conducted semistructured virtual interviews with 21 family caregivers and 2 focus groups with 7 PLWD to explore their experiences during the COVID-19 pandemic [29]. Findings showed a substantial need for in-home care strategies to manage stress among PLWD and care partners, specifically strategies not reliant on in-person training or interaction with people outside the home. Cumulatively, these studies provide a preliminary understanding of the in-home care stress management needs of PLWD and their care partners, as well as the potential of 1 stress management tool for PLWD; however, the knowledge gained is not sufficient to fully understand the stress experiences of dementia caring dyads, or the potential of a more comprehensive dyadic stress management toolkit. In this way, these studies provide a foundation for the proposed study.

## Methods

### Study Design

This study consists of 2 phases. The first phase will address the *discover*, *define*, and *design* stages of HCD using qualitative, semistructured focus groups with dementia caring dyads to develop a stress management toolkit prototype (aim 1). The second phase will address the *validation* step of HCD by user testing the prototype, and exploring the feasibility of collecting stress-related outcomes (aims 2 and 3). The trial is registered in ClinicalTrials.gov (NCT05465551).

### Phase 1: Discover, Define, and Design (Aim 1)

#### Overview

Phase 1 will use qualitative focus groups with dementia caring dyads to *discover* and *define* the experiences, perceptions, and preferences of PLWD and their care partners regarding stress and stress management. Their preferences and recommendations regarding key components and format of the stress management toolkit will be explored to *design* the prototype.

#### Phase 1 Participants and Recruitment

PLWD and their primary, informal care partners will be recruited together as participant dyads [30]. Inclusion criteria for participants with dementia are as follows: (1) age 60 years and over with a diagnosis of dementia of any type; (2) able to express self verbally; and (3) English speaking. We will purposively sample PLWD who experience some degree of stress, operationalized as demonstrating at least two symptoms listed on the Neuropsychiatric Inventory within the most recent 4 weeks as reported by the PLWD or their care partner [31-34]. Exclusion criteria for participants with dementia are as follows: (1) has a hearing or visual impairment that limits their ability to participate in the screening process or to participate in a focus group. Inclusion criteria for care partner participants are as follows: (1) age 21 years and older; (2) identify as a primary care partner of someone with dementia; and (3) English speaking [32,35]. Exclusion criteria for care partner participants are as follows: (1) has a hearing or visual impairment that limits their ability to participate in the screening process or to participate in a focus group. Dyadic eligibility criteria include the following: (1) both the PLWD and care partner reside in the same household or personal residence in the community; (2) dyad has lived together for at least one month; and (3) dyad has telephone or internet access. Dyads will be excluded if they reside in assisted living or other long-term care settings.

The projected sample size for this phase is 12-16 dyads based on similar prior studies, but up to 25 dyads will be enrolled if necessary to reach data saturation [35,36]. Dyads will be recruited through several regional and national dementia and caregiver community support organizations that provide a range of services, resources, and referrals to PLWD and their caregivers. Recruitment organizations provide support through in-person, as well as virtual educational offerings, advocacy events, and social engagement activities. Recruitment flyers and study information will be distributed through in-person and virtual events. The research team will attend in-person and virtual offerings to provide more detailed information regarding the study. Interested individuals will be able to contact the research team directly to learn more about the study and to determine eligibility. Designated staff members at recruitment organizations will also collect names/contact information for individuals that express interest and agree to be contacted directly. An eligibility determination form with the criteria outlined above will be completed by a research team member (MH) for all interested individuals that are contacted regarding study participation.

#### Phase 1 Consenting Procedures

Eligible participants will provide consent verbally to participate in phase 1 of this study. To complete the consent, participants will review a study information form with a research team member. The study information will be sent by email, by postal mail, or reviewed verbally with potential participants depending on their preference. After reviewing the information sheet, participants will then be asked verbally if they wish to participate in the study. Both members of the dyad must provide verbal consent. The researcher obtaining consent will use an inclusionary, person-centered approach throughout the discussion by incorporating several strategies to enhance the PLWD’s ability to remain engaged and empowered throughout the consent discussion [37,38]. This will include strategies such as assessing for verbal and nonverbal cues indicative of the person’s interest and degree of engagement in the conversation, by offering multiple opportunities for the PLWD to ask questions, by using language that is understandable to the PLWD, by frequently restating the key features of the study in different ways, by asking the PLWD to restate the key features throughout the discussion, by offering information in multiple ways (ie, written, verbal, visual examples), by offering breaks in the discussion, by offering to schedule a follow-up call to complete the discussion at another time.

#### Phase 1 Focus Group Guide and Data Collection

Four focus groups (n=3-4 dyads/group) will be held virtually over Zoom (Zoom Video Communications, Qumu Corporation) or in-person, dependent on safety and participants’ preference. Focus groups were selected as they allow for collaborative idea generation to evolve more quickly through group discussion, which is an essential component of the *design* step of HCD [27,39]; however, individual or dyadic interviews will be used if scheduling focus groups with dyads becomes a challenge or if needed to optimize engagement for PLWD. A trained research team member (MH) will facilitate the focus groups using a semistructured focus group guide, which will concentrate on dyads’ experiences and perceptions regarding stress, stress management, and the toolkit prototype. Participants will be asked to discuss their experiences with stress at home and how they manage stress currently. The focus group facilitator will then present a visual demonstration of what a tangible stress management toolkit could entail; for example, tools such as dementia-friendly music devices, social robot pets and dolls, acupressure and massage tools, weighted blankets and garments, prompted journals, aromatherapy, and bright light therapy devices. The group will be asked if these tools seem relevant or potentially useful to them and if the tool seems like it could fit into their daily lives at home. They will be asked to describe other tools that could be a good fit for the toolkit. The group will then be asked how they would like a stress management toolkit to look, how they would like it to be delivered to them, and what additional information they would like to have included with the toolkit. The focus group guide will include questions directed at PLWD and care partners. The topics discussed may be modified based on findings that emerge throughout the study.

Focus groups are anticipated to last about 90 minutes and will be audio recorded and transcribed using Zoom’s virtual conferencing platform. A trained research team member will take detailed notes using a semistructured note-taking guide. Immediately after each focus group session, the notetaker and the group facilitator will develop postgroup summaries and debrief notes that highlight key points from the group [40,41]. If necessary, additional focus groups or individual interviews will be held if it seems that the perspectives of PLWD or care partners or both are not fully reflected in the dyadic focus groups, or if data saturation is not reached to fully inform the development of the toolkit prototype.

Information pertaining to the sociodemographic and diagnosis characteristics (such as age, race and ethnicity, gender, education, marital status, dementia type, duration of diagnosis, relationship between dyad composition, duration of having lived together, residence geographical location [urban, suburban, or rural]) of the dyad will be collected prior to scheduled focus groups. To further describe the degree of stress of the sample, information will also be collected pertaining to concepts such as the perceived stress of participants with dementia and care partners, and dyadic strain (as perceived by PLWD and care partners). Psychometrically sound measurement tools will be used to obtain this information (Table 2), which will be collected using questionnaires completed by phone, hardcopy, or electronically depending on participant preference. A care partner–specific questionnaire as well as a PLWD-specific version questionnaire will be completed by each dyad. Each questionnaire is anticipated to take approximately 10-15 minutes to complete. Participants will complete questionnaires before their scheduled focus group electronically, by hardcopy, or by phone depending on their preference.

**Table 2:** Measures to be collected to describe degree of stress of participants.

Operationalized measure	Measurement instrument (number of items)	Psychometric properties	Source of completion
Perceived stress of PLWD^a^	Perceived Stress Scale: reported by PLWD (10)	Cronbach α=.74 Convergent validity established with the Geriatric Depression Scale [42-44]	PLWD
Perceived stress of care partner	Perceived Stress Scale: reported by care partner (10)	Cronbach α=.80-.84 Convergent validity established with the Geriatric Depression Scale [42-44]	Care partner
Dyadic strain	Dyadic Relationship Scale: reported by PLWD (10)	Cronbach α=.84-.86 Construct validity established through confirmatory factory analysis [45]	PLWD
	Dyadic Relationship Scale: reported by care partner (11)	Cronbach α=.84-.89 Construct validity established through confirmatory factory analysis [45]	Care partner

^a^PLWD: people living with dementia.

#### Phase 1 Data Analysis

Stress-specific measures to describe the sample will be scored for each participant using measurement scoring guidelines [42,43,45]. Descriptive statistics (means, SDs, medians, frequencies, percentages) will be calculated to describe the sociodemographic, diagnosis, and stress-specific characteristics of the sample.

This study will use a rapid qualitative analysis approach with purposeful data reduction activities to facilitate ongoing and iterative data analysis [40,41]. All groups will be recorded and transcribed using Zoom’s transcription function. Immediately after each group, the first author will code notes and summaries by identifying patterns, diverging points, critical quotations, and key points. Prior research on intervention development and HCD pertaining to discovering and defining the problem (ie, stress, stress management) and designing the prototype (eg, characteristics, components, delivery) will be used to generate initial categories for the analysis and will provide a scaffold to build off for the coding process [19,20,27,46]. A list of initial codes, definitions, and exemplar quotations will be kept in a codebook. At least one other analyst will listen to the audio recording or refer to transcripts, review notes, and summaries, and then add to and modify the codebook developed by the first author. Differences in coding will be indicated using highlighting and comments to be discussed among analysts during weekly meetings to reach consensus regarding codes and code groups (or themes) [41]. Data will be compared within and across focus groups iteratively throughout the study. Initial codes and themes will be presented to at least two participant dyads. They will provide feedback and participate in discussions with the research team to finalize the findings, which will inform development (eg, specific tools, components, features, user guides, delivery techniques) of the prototype. Multiple phases of feedback may be needed prior to finalizing the prototype [19,27,47].

### Phase 2: Validate (Aims 2 and 3)

**Figure 1:**
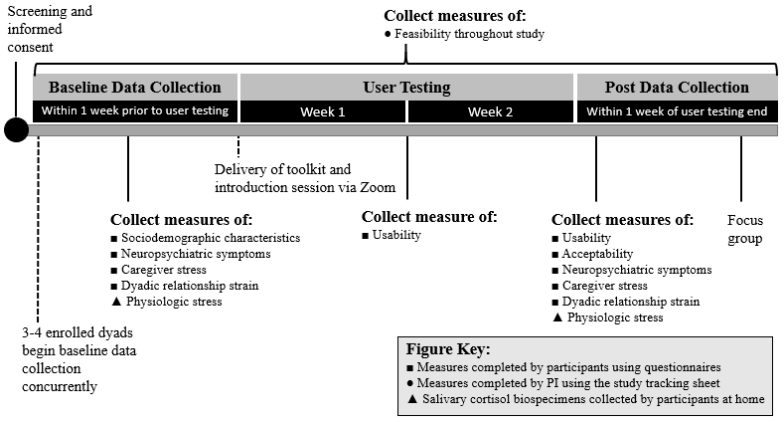
Study overview. PI: principal investigator.

Phase 2 will address the *validate* step of the HCD process (Table 1). A total of 10 dyads (who were not involved in phase 1) will use the prototype toolkit for 2 weeks and measures of usability, feasibility, acceptability, and stress-related outcomes will be collected (Figure 1).

#### Phase 2 Participants and Recruitment

Similar eligibility criteria will be used in phase 2 as were used in phase 1 with few additional criteria to address contraindications to the collection of salivary cortisol biospecimens. In addition to the phase 1 criteria, participants will be excluded if they (1) currently receive cytokine-based therapy; (2) currently receive radiation therapy to the salivary glands or thyroid; (3) are diagnosed with Cushing or Addison disease. Dyads will also be excluded in phase 2 if they participated in phase 1. The same recruitment strategy used in phase 1 will be used in phase 2 as well. Ten dyads was selected as the projected sample based on prior studies that have used HCD with this population [26,27], but up to 20 may be enrolled if data saturation is not reached.

#### Phase 2 Consenting Procedures

All participants will provide signed consent to participate in this phase of the study. Eligible dyads will be sent an informed consent form electronically or as a hardcopy depending on their preference. Electronic forms will be distributed by email via an electronic-consenting platform and hardcopies will be sent by U.S. Mail. Similar steps used in phase 1 will be followed in phase 2 to obtain consent with the exception that both members of the dyad will provide signed consent, as opposed to verbal consent.

#### Phase 2 User Testing Procedures

Dyads will receive a toolkit and toolkit user guide by mail after collection of baseline data. Dyads will participate in an introduction session with a trained research team member via Zoom or phone to review how to use the toolkit. Dyads will use the toolkit in the home for 2 weeks. A 2-week duration was selected based on prior home-based studies with this population that have used HCD [48,49]. Dyads will be encouraged to use at least one tool from the toolkit at least once a day to manage day-to-day stress and to mitigate the risk of negative outcomes related to excess stress.

#### Phase 2 Measures

##### Aim 2

Usability will be measured by examining the frequency of use of the toolkit by participants. At the end of weeks 1 and 2 of user testing, dyads will complete a brief questionnaire to indicate tools that were used and by whom (ie, PLWD or care partner), number of days the toolkit was used over the past week, and each participant’s general response to the toolkit. The toolkit will be considered to have a high degree of usability if participants use it on average 9 or more days/14-day intervention period [28,50]. *Feasibility* will be measured throughout the study by examining several measures such as study enrollment and withdrawal rates, and adverse effects and injuries [51]. The prototype will be considered feasible if enrollment rate is 50% or more, withdrawal rate is 25% or less, and no adverse events or injuries were reported [52-54]. *Acceptability* will be measured at the end of week 2 of user testing. Dyads will complete item scales pertaining to what they found most beneficial and satisfying regarding the toolkit, and challenges experienced when using the toolkit. An acceptability survey will be developed for this study that will be modified from prior tools used to measure acceptability of nonpharmacologic dyadic-focused interventions for patients with chronic conditions and their care partners [55,56].

*Usability*, *feasibility*, and *acceptability* will be further explored through qualitative focus groups (3 groups in total; n=3-4 dyads/group) convened after participants have completed posttest questionnaires. Similar procedures used for focus groups in phase 1 will be used in phase 2 to explore the usability, feasibility, acceptability of the toolkit, and participants’ recommendations regarding modifications and refinements needed to improve the toolkit. Throughout the study, we will also keep a tracking sheet of the specific tools and cost of each toolkit delivered to participants in phase 2. We will ask questions pertaining to cost during the focus groups to begin to explore participants’ perceptions of cost and willingness to pay for such a toolkit out-of-pocket as a component of acceptability [57].

##### Aim 3

*Stress-related participant-reported outcomes* (eg, neuropsychiatric symptoms of dementia, caregiver stress, dyadic relationship strain) measured using psychometrically sound measurement tools [32,42,45] and *salivary cortisol biospecimens* of participants with dementia and care partners [58] will be collected at baseline and after user testing (Figure 1 and Tables 3 and 4). Outcome measure collection will be feasible if the measure can be collected in 80% or more of participants at baseline and after data collection timepoints.

**Table 3:** Phase 2 outcome measures for aim 2.

Outcome	Measure	Measurement	Data collection tool
Usability	Frequency of toolkit use	How many days in the past week was the toolkit used by the participant with dementia/care partner? (range 0-7 days)	Questionnaire
Feasibility	Enrollment rate	Percentage of participants enrolled	Study tracking sheet
	Withdrawal rate	Percentage of participants that withdraw	
	Adverse events/injuries	Number of adverse events and injuries	
Acceptability	Satisfaction	Toolkit satisfaction scale (1=not satisfied at all to 5=very satisfied)	Questionnaire
	Perceived benefit	Toolkit benefit scale (1=not at all beneficial to 5=very beneficial)	

**Table 4:** Phase 2 outcome measures for aim 3.

Outcome	Measurement	Psychometric properties	Data collection tool
Neuropsychiatric symptoms of dementia	Neuropsychiatric Inventory-Questionnaire [34]	Cronbach α (range)=.71-.88 Percentage agreement between raters: 93.6%-100% Test-retest reliability range (r)=0.79-0.86 [34,59,60]	Questionnaire
Caregiver stress	Perceived Stress Scale [43]	Cronbach α (range)=.75-.82 External validity established through CFA^a^ [42,44]	Questionnaire
Dyadic relationship strain	Dyadic Relationship Scale [45]	Cronbach α (range)=.84-.89 External validity established through CFA [45]	Questionnaire
Physiologic stress	Salivary cortisol biospecimens	N/A^b^	Oral swab

^a^CFA: confirmatory factor analysis.

^b^N/A: not applicable.

#### Phase 2 Data Collection

##### Aim 2

Feasibility data will be collected by the research team throughout the study using a tracking sheet stored on a secure institutional server. Usability and acceptability data will be collected using questionnaires completed electronically, by hardcopy, or verbally by phone in accordance with participant preference. Similar data collection procedures described in phase 1 will be used to collect questionnaire data in phase 2. Sociodemographic and diagnosis characteristics of the dyad will also be collected using a questionnaire. Similar qualitative data collection procedures used in phase 1 will be used to collect focus group data in phase 2.

##### Aim 3

*Stress-related participant-reported outcome measures* will be collected using questionnaires completed electronically, by hardcopy, or verbally by phone, depending on participant preference. *Salivary cortisol biospecimens* will be collected by participants using oral swab kits. Participants with dementia and care partners will each provide 10 samples in total. Five samples (per participant) will be collected over the course of 1 day at baseline and the remaining 5 will be collected over the course of 1 day after data collection. Participant dyads will receive sample kits by postal mail at least one week prior to the 2 data collection timepoints (baseline and after). Each dyad will participate in a telephone or Zoom session with a research team member to review how to collect saliva samples prior to the scheduled data collection timepoint. During this session, participants will be asked to not complete the sample collection if any of the following are present on the day of data collection: (1) a fever >101°F, (2) upper respiratory infection with nasal drainage, and (3) inflammation of the throat. As appropriate, an alternative day will be identified if any of these symptoms are present, or the data collection will be canceled and documented as incomplete in the study database.

A research team member will be available by phone or Zoom for participants to ask questions and receive support during the collection of salivary samples. Participants will also be able to access video instructions provided by Salimetrics [61]. Detailed methods for saliva collection and handling can be found online [62]. In brief, each Salimetrics kit contains 1 oral swab, 1 swab storage tube, and sample collection instructions. Participants peel back the protective package around the swab and place it under their tongue for 2 minutes after which it is placed into the prelabeled storage tube. The swab-containing storage tube is then placed in the refrigerator. On each measurement day (baseline and after) each participant will provide a saliva swab at waking, 30 minutes after waking, 60 minutes after waking, 1 hour before or after dinner, and 1 hour before bedtime for a total of 5 swabs per time point, per participant [63]. Care partners may assist the PLWD in collecting their samples. The swabs will be shipped to the corresponding institution’s biomarker laboratory using a prepaid package with a small ice pack, which will be picked up by FedEx personnel for delivery. Samples will be immediately frozen and stored at the biomarker laboratory at –20°C until assayed by a Salimetrics-certified laboratory. Collection and storage methods follow the assay manufacturer (Salimetrics) recommendations and have been validated [64].

#### Phase 2 Data Analysis

##### Aim 2

Descriptive statistics (means, SDs, frequencies, percentages) will be calculated for measures of usability, feasibility, and acceptability. Similar rapid qualitative analysis methods that were used in phase 1 will be used to analyze phase 2 focus group data.

##### Aim 3

Percentage of participants with data collected will be calculated for each outcome measure at each data collection timepoint. To further describe the study sample, the Neuropsychiatric Inventory, Perceived Stress Scale, and Dyadic Relationship Scale measures will be scored for each participant according to measurement scoring guidelines at each timepoint and group means (SDs) will be calculated. We will not assay the salivary cortisol specimens in this study, but will do so as a secondary analysis in a future study.

### Ethics Approval

This study was approved by the Duke Health System Institutional Review Board (HUM00186832).

## Results

This study received IRB approval in August 2022 and is anticipated to be completed by July 2024. Expected outcomes of phase 1 are a working prototype of a dyadic stress management toolkit for PLWD and their care partners. Phase 2 will yield preliminary data to support the feasibility and acceptability of the toolkit, as well as data to inform the design (eg, measurement selection, recruitment, data collection methods) of a future study to examine efficacy. The goal is that by using an HCD approach that incorporates stakeholder engagement at the onset of development, this intervention will be more applicable and acceptable to families living with dementia. Exploring feasibility and acceptability in the early stages of intervention development will help determine whether costlier efficacy testing is warranted [65]. Data generated from this study will act as a stepping stone in the development of a stress management intervention for dementia caring dyads that has an increased likelihood of being implemented and sustained in the future.

## Discussion

### Expected Findings

The need for home-based stress management interventions for PLWD and their care partners was amplified by the COVID-19 pandemic [29,66]. Tangible stress management tools exist that are passive in nature (eg, low user burden, minimal training required) and safe for use by older adults, but there remains a paucity in research focused on the use of such tools by PLWD and their care partners. Furthermore, no prior studies have focused on combining multiple tangible tools to promote stress management among community-dwelling dementia caring dyads. Findings will expand state of the science by developing and user testing a tangible stress management toolkit for dementia caring dyads using an HCD approach. Qualitative findings pertaining to usability, feasibility, and acceptability will elucidate areas to refine the toolkit. In addition, insights relating to participants’ attitudes toward the cost of toolkits will provide preliminary information pertaining to the scalability of this intervention. Examining the feasibility of collecting several stress-related outcome measures will also inform measurement selection in a future efficacy study. This study will yield a working prototype of the stress management toolkit, as well as preliminary data to support the feasibility and acceptability of the intervention.

This study incorporates several innovative components including the use of HCD. HCD involves identifying problems, co-designing solutions with key stakeholders, and user testing solutions with end users early in the intervention development process. HCD is a promising approach to identifying solutions for families living with dementia as many nonpharmacologic interventions have demonstrated limitations in broader implementation and sustainability in this population. A second innovation is the inclusion of salivary cortisol samples as a biologic measure of stress. Few nonpharmacologic intervention studies have examined intervention effects from a physiologic stress-response perspective. This study will help determine whether salivary cortisol is a feasible outcome to include in a future study to determine efficacy of the toolkit intervention, as well as other intervention studies focused on community-dwelling dementia caring dyads. Strengths of this study are the use of predefined measures of usability, feasibility, and acceptability; a national recruitment strategy; multiple stakeholder engagement strategies; and remote and in-home data collection methods. Offering multiple ways to engage in this study provides a more equitable approach by circumventing barriers to support service access and research participation (eg, rurality, transportation, limited internet access).

Potential limitations of this study include issues relating to recruitment and measurement. Stress and stress management experiences and preferences are dependent on individual customs, cultures, and historical and social contexts. Thus, the specific components of the toolkit will depend greatly on the individual experiences of participants in phase 1. Although we will use a national recruitment strategy with the intent of recruiting a diverse sample in terms of sociodemographic, disease, and caregiving characteristics (eg, race/ethnicity, rurality, dementia type, relationship between dyad), it is likely that the prototype will be more relevant to some dyads compared with others. Throughout the design process we plan to incorporate multiple opportunities to personalize the toolkit based on individual and dyadic preferences to enhance generalizability and applicability. In terms of measurement limitations, many of the proposed measures in phase 2 are based on self or proxy report, which carries an innate potential for biased responses. This is particularly significant for measures of usability and acceptability of the toolkit as prior studies demonstrate discrepancies in perceived versus actual use of self-care interventions, and an increased risk of providing socially acceptable responses on satisfaction surveys [67,68]. To address this concern, we will include verbiage in questionnaire directions and verbally encourage participants to provide honest responses to these measures. We will reassure participants that they will not be judged or treated differently based on the responses they provide. The small sample for this study is congruent with feasibility study design guidelines; however, findings will be limited in terms of generalizability and future testing will be needed to determine efficacy. These limitations notwithstanding, this study is well positioned to provide the necessary data to inform the design of a successful pilot efficacy trial in the future.

### Future Directions

If findings demonstrate feasibility and acceptability of the toolkit, a critical next step will be determining efficacy of the toolkit intervention for improving stress-related outcomes through a larger randomized controlled trial [69]. Future research may also explore the cost-benefit of the toolkit, as well as optimal “dose” or amount of toolkit use needed to yield clinically significant effects. It will also be important to examine how contents and delivery of the toolkit may be tailored for dyads based on their individual and cultural preferences. Findings from this feasibility study will provide necessary data to inform measurement selection, data collection methods, and recruitment capacity for future studies.

### Conclusions

PLWD and their care partners are in desperate need of home-based strategies to reduce stress and promote well-being. This study uses an HCD approach to develop and user test a tangible stress management toolkit *with* and *for* dementia caring dyads. This study will be the first to combine several stress management tools into a comprehensive toolkit designed for use by PLWD and their care partners. Using an HCD that involves stakeholder engagement at the onset of development will increase the applicability of the intervention to the target population. By examining feasibility and acceptability early in the development process, this study will act as a foundation for future testing.

## Appendix

Multimedia Appendix 1 Summary of peer review from the Research Education Core of the Duke/University of North Carolina Alzheimer’s Disease Research Center (ADRC).

## Abbreviations

HCD: human-centered design
PLWD: people living with dementia

## Data Availability

Data sharing is not applicable to this article as no data sets were generated or analyzed during the preparation of this study protocol.
