# Supplementary material for: Development of a Home-Based Stress Management Toolkit for Dementia Caring Dyads: Protocol for a Pilot Intervention Development and Feasibility Study
Source: JMIR Res Protoc. 2022 Dec 14;11(12):e43098. doi: 10.2196/43098 (PMC9798268; doi:10.2196/43098)
Supplement: Multimedia Appendix 1 [file resprot_v11i12e43098_app1.pdf]

Duke Neurosurgery

Duke, UNC & Me
Preventing Alzheimer's Disease Together

March 25, 2022

Melissa Harris, PhD, RN
National Clinician Scholars Program Postdoctoral Fellow 2021-2023
Duke University School of Nursing
Durham VA Health Care System
Durham, North Carolina 27705

Re: Study application, "*Development of a home-based stress management toolkit for dementia-caring dyads*"

Dear Dr. Harris:

I am pleased to inform you that your proposed project has been selected for funding by the Research Education Core of the Duke/UNC Alzheimer's Disease Research Center (ADRC). This selection is based upon the merits of the research proposed in your application, as well as your promise to advance our shared mission of translating research advances into improved prevention, diagnosis and care for patients and families living with Alzheimer's disease and related dementias (ADRD).

Below you can find your summary statement. Although individual scores and comments from your three reviewers are not available, the rubric below represents the summary statement from the six ADRC Executive Committee members. This document incorporates all reviewer comments, as well as additional input from our ADRC Executive Council.

We very much look forward to your work on this important topic and to collaborating with you through the ADRC REC Scholars Program.

Sincerely,

Kyle M. Walsh

Kyle M. Walsh, PhD
Associate Professor, Departments of Neurosurgery and Pathology
Director, Division of Neuro-epidemiology
Co-leader, Duke Cancer Institute Program in Neuro-oncology
Senior Fellow, Duke Center for the Study of Aging and Human Development
Duke University School of Medicine

DUMC Box 3050 | 442 MSRB-1, 571 Research Drive | Durham, NC 27710
Phone (919) 684-8732 | Email: Kyle.Walsh@Duke.edu

Duke/UNC ADRC REC Scholars, 2022 Scoring Summary
Reviewer Names: Whitson, Roberts, Roberson
Project title: Development of a home-based stress management toolkit for dementia-caring dyads
PLEASE LET US KNOW IF YOU HAVE ANY POTENTIAL CONFLICTS OF INTEREST OR OTHER CONCERNS ABOUT THIS PROPOSAL: *none noted*

REVIEW SECTION

NOTE: All comments in the following section may be provided to the applicants.

**Criterion**	**Score (1-9); 1=exceptional, 9=poor**	**Comments**
Overall impact	2	Impact was assessed as high given strong component scores and a consensus that this project has the ability to develop innovative interventions for ADRD patients/caregivers and to measure their utility both qualitatively and quantitatively. The study is conceptually ambitious but feasible in scope. Strong mentorship and dedication to the field elevate the impact of the proposal.
Significance	2.3	Interventions are very much needed to reduce stress among families living with dementia. The use of a home-based intervention that places minimal burden on care partners is highly significant, particularly for rural-dwelling families. The choice to conduct a dyadic intervention and to utilize a human-centered design approach prioritizing stakeholder engagement elevates the translational significance of the proposed work.
Innovation	1.3	Low-cost and low-burden stress-reduction interventions that can be used in the home setting are innovative, as is the iterative toolkit development process that is likely to maximize user uptake. Inclusion of a cortisol biomarker is innovative and somewhat “outside the box” for a study such as this, and it has the potential to bridge gaps in

		psychosocial research with biomarker/pathology research.
Approach	1.7	Aim 1 will convene a 4 focus groups to explore perfor needs assessment and to identify key features of a stress management toolkit. Enrollment is feasible, qualitative analysis is well-described, and team is well-versed in the study techniques.<br>Aim 2 will assess feasibility and acceptability of prototype toolkit in 10 dyads with questionnaire and focus group feedback to assess these measures. Some concerns regarding feasibility of delivering toolkit to 10 dyads simulataneously so that focus groups can include multiple dyads at end of week 2. Likely that focus groups can be conducted with 3-4 dyads at end of week two, meaning that three different delivery dates can be used and mitigating this issue.<br>Aim 3 will assess feasibility of collecting stress-related outcome measures in dyads using combination of validated neurospych measures and cortisol biomarkers. Standard issues relating to patient-reported outcomes in patients living with dementia apply, but are mitigated by the innovative inclusion of an additional biomarker of stress (salivary cortisol). Obtaining usable cortisol samples from 80% of cohort is not likely, but this is a feasibility study that will guide and help power future research. A qualitative measure of how dyads perceived the saliva collection is advised.
Investigators	1	Exceptional candidate with a strong career outlook as a nurse-scientist. T32 training during grad school and current position in the National Clinical Scholars Program will synergize with REC training.
**Programmatic Questions**	**Yes/No**	

Meets the goals of the REC?<br>• Investigates AD/ADRD?<br>• Supports a junior investigator who does not yet have R01-level funding?<br>• Involves Duke/UNC-CH/ECU/NCCU/UNCP collaborators, or targets patients in the ADRC catchment area?	• **Yes**<br>• **Yes**<br>• **Yes**	
Potential for future funding	1	Application deemed highly likely to support future clinical investigator K award application to NIA, NINDS, NIMH
Mentorship	1	Strong letter from Nikki Hastings (Duke and VA geriatrician, Director of the Durham VA Center of Innovation to Accelerate Discovery and Practice Transformation). Candidate has strong working relationship with mentor, including co-published manuscripts.
Career Development Plan	1	High potential to be a successful nurse-scientist. Formerly a T32 predoctoral trainee at UM, currently a fellow in Duke's National Clinician Scholars Program.
Multidisciplinary research?	1.7	Research focuses on non-pharmacologic interventions to reduce stress and improve well-being of people living with dementia and their care partners. Will mix qualitative and quantitative assessments, including biomarkers of stress-response.
Proposed research supports a future career in ADRD?	1	Has strong grasp of the necessary training and productivity steps necessary to transition to career independence. Moving from T32 to NCSP to REC Scholar will put her on-track to successfully compete for K award and faculty positions in dementia care.
